# ChatDashboard: A Framework to collect, link, and process donated WhatsApp Chat Log Data

**DOI:** 10.3758/s13428-023-02276-1

**Published:** 2023-12-21

**Authors:** Julian Kohne, Christian Montag

**Affiliations:** 1 Department of Molecular Psychology, Institute of Psychology and Education, Ulm University, Ulm, Germany; 2 GESIS – Leibniz Institute for the Social Sciences, Department of Computational Social Science, Unter Sachsenhausen 6-8, 50667 Cologne, Germany

**Keywords:** ChatDashboard, WhatsR, DashboardTester, WhatsApp, Data donation, Relationships, Computer-mediated communication

## Abstract

In this paper, we present ChatDashboard, a framework for collecting, linking, and processing donated WhatsApp chat log data. The framework consists of the WhatsR R package for parsing, anonymizing, and preprocessing donated WhatsApp chat logs, the ChatDashboard R Shiny web app for uploading, reviewing, and securely donating WhatsApp chat logs, and DashboardTester, an automated script for testing the correct setup of the framework by simulating participants. With ChatDashboard, researchers can set up their own data collections to gather transparently donated WhatsApp chat log data from consenting participants and link them to survey responses. It enables researchers to retrospectively collect highly granular data on interpersonal interactions and communication without building their own tools from scratch. We briefly discuss the advantages of donated WhatsApp chat log data for investigating social relationships and provide a detailed explanation of the ChatDashboard framework. Additionally, we provide a step-by-step guideline in the supplementary materials for researchers to set up their own data donation pipelines.

Measuring social relationships has always been an essential task across all domains in academic psychology. Having close and meaningful social relationships is one of the most pervasive human needs (Baumeister & Leary, 1995), and consequently has a large influence on our mental (e.g., Bertera, 2005; Cohen & Wills, 1985; Cooper et al., 2021; Horwitz et al., 1998; Kawachi & Berkman, 2001; Lakey et al., 1994; Santini et al., 2015; Vanderhorst & McLaren, 2005) and physical health (Berkman & Kawachi, 2000; Cacioppo & Cacioppo, 2014; Cohen et al., 1997; Holt-Lunstad & Smith, 2012; J. S. House et al., 1988; Kiecolt-Glaser et al., 2010), resilience (Afifi et al., 2016; Fuller-Iglesias et al., 2008; Southwick et al., 2011), and life satisfaction (Haller & Hadler, 2006; Holder & Coleman, 2009). Social relationships at least partly shape who we are and how we see ourselves (Aron et al., 1995; Klimstra, 2013; Mattingly et al., 2014, 2020; McIntyre et al., 2017; Slotter et al., 2010), our values (Biber et al., 2008; Podolskiy, 2012), what we perceive as normative (Bicchieri & Mercier, 2014; B. R. House, 2018; Kohne et al., 2019; Neumann, 2008), and how we behave (Cialdini & Goldstein, 2004; Latané, 1981). As such, understanding and measuring social relationships, from parent–child interactions (Funamoto & Rinaldi, 2015; Kerns et al., 2000; Peisah et al., 1999; Saunders & Schuchts, 1987) to romantic relationships (Hendrick & Hendrick, 2003; Langeslag et al., 2013; Overbeek et al., 2007; Rubin, 1970; Sternberg et al., 2006) and friendships (Hawthorne & Griffith, 2000; Helmi et al., 2017; Nielsen et al., 2000; Sharabany, 1994), or larger social networks including colleagues and acquaintances (de la Haye et al., 2010; Moore, 1990; Rosenquist et al., 2010; Ueno, 2005; Zagenczyk et al., 2013), has always been a key area of psychology and related disciplines. Over the past decades, a large variety of methods have been used to measure social interactions and communication in social relationships, each with their own unique advantages and disadvantages.

In the following sections, we will outline why chat log data, and more specifically WhatsApp chat log data, can effectively complement existing data collection procedures. To this end, we will first discuss the advantages and disadvantages of established methods for measuring interpersonal interactions and communication and compare them to the features of WhatsApp chat log data. Subsequently, we will argue that WhatsApp chat logs provide a promising opportunity for researchers to collect highly detailed data on interpersonal interactions unobtrusively and retrospectively. We underline that these data can reduce many of the potential biases that other methods for collecting data on interpersonal interactions and communication have, while also being more scalable to larger data collections. However, we also point out the complexities of WhatsApp chat logs as research data and why these complexities present an obstacle to using WhatsApp chat log data for most social scientists.

Consequently, we introduce ChatDashboard—a framework to facilitate the collection and processing of donated WhatsApp chat log data for social scientists. The framework consists of the WhatsR R package as a backend for parsing and preprocessing donated WhatsApp chat logs, the ChatDashboard R Shiny web app for uploading, reviewing, and securely donating WhatsApp chat logs, and DashboardTester as an automated script for simulating participants for testing purposes. ChatDashboard enables researchers to build their own data donation pipelines for collecting transparently donated WhatsApp chat logs from their participants and link them to additional data sources, such as survey responses. In the main sections of this paper, we explain the ChatDashboard framework and the decisions shaping its design. In the supplementary materials, we provide a step-by-step guideline for researchers to implement it in their own data collections.

## Established measures of interpersonal interactions and communication

According to Miller (2014, p. 50–61), at least five major paradigms have traditionally been used for gathering data about interpersonal interactions and communication in interpersonal relationships, and all come with unique advantages and disadvantages. For every research project, scientists thus need to make strategic decisions regarding which data collection paradigm would be the most appropriate to answer their research questions, as well as which benefits they are willing to sacrifice for which drawbacks.

While self-report measures grant insight into people’s unique, subjective experiences, they are prone to *subjectivity* (Gute et al., 2008; Chabot et al., 2013; Wiederman, 2004, as cited in Miller, 2014) and *social desirability biases* (Schick et al., 2014; Follingstad & Rogers, 2013; Fisher, 2013, as cited in Miller, 2014). In addition, they may elicit *observer-expectancy effects* (Kintz et al., 1965; Rosenthal, 1963) or *reactivity* (French & Sutton, 2010; McCambridge et al., 2014) when participants align their responses with what they assume researchers to expect. Moreover, people often simply do not accurately *remember* how they behaved in the past (Aicken et al., 2013; Grote & Frieze, 1998; Mitchell, 2010, as cited in Miller, 2014).

Experiments have the unique advantage of establishing a causal link between two variables and can reduce *observer-expectancy effects* by deceiving participants about the nature of the study (Miller, 2014, p. 53). However, they are also costly, time- and personnel-intensive, inconvenient for research participants, and only allow researchers to observe participants in artificial settings with their full awareness of being evaluated. This raises concerns about biases in terms of *reactivity* and *mundane realism* (Berkowitz & Donnerstein, 1982; Miller, 2014, p. 53).

Observational studies allow researchers to gather data in realistic settings without concerns about *mundane realism*, *reactivity*, *social desirability biases*, or *observer-expectancy effects* (Miller, 2014, p. 59-60). On the other hand, participants in observational studies cannot consent before they are observed and can only be observed in naturally occurring, public situations. This can make them unfeasible, personnel- and time-intensive, and might yield only a small number of cases (cf. Carey, 2010, as cited in Miller, 2014, p. 60).

Physiological measures such as heart rate (Mosley & Laborde, 2022; Schiweck et al., 2019) or loudness and frequency of voice (Hussain et al., 2023) have the advantage that they are less distorted by social desirability, subjectivity, or observer-expectancy effects, and can also pick up subtle or unconscious signals that are otherwise difficult to quantify (Miller, 2014, p. 60). While these methods were restricted to the laboratory in the past, the necessary equipment has become more portable and less intrusive in recent years. Smartphone-based data collection methods in particular have improved substantially (Harari et al., 2016; Sariyska & Montag, 2023; Timmons et al., 2017; Wrzus & Neubauer, 2023). Nevertheless, the awareness of being observed alone may already bias such measurements (French & Sutton, 2010; McCambridge et al., 2014; Montag et al., 2016).

Finally, archival studies rely on traces of human behavior to gain insights into behavioral patterns. Marriage records, governmental statistics, or census data offer the opportunity to study populations of people retrospectively without them being aware that their behavior is being analyzed (Miller, 2014, p. 61). Their disadvantage is that researchers have no control over their sample or their variables of interest, data tend to be very sparse, and the results often only apply to previous generations (Miller, 2014, p. 61).

## Instant messenger data

In contrast to the previously mentioned paradigms for collecting data about interpersonal interactions and communication, instant messaging services offer social science researchers a novel addition to their toolboxes. Most instant messengers essentially log interpersonal interactions passively while the application is being used, and can thus provide a detailed report of all interactions between two or more people within the respective conversation. These data can be requested by users, and donated directly to researchers. Such data donations ensure that research participants consent to their data being used, ensure transparency about the content of donated data, and make the research process more independent from restrictions and regulations of large platform providers (see Boeschoten et al., 2020; Ohme et al., 2020; Ohme & Araujo, 2022; and Breuer et al., 2022 for an in depth discussion). However, working with instant messenger data is a tedious process due to the many practical challenges with respect to incentivizing participants, collecting data, preprocessing it, ensuring informed consent, anonymization, and high heterogeneity of data (see also Kohne et al., 2022). For this reason, a substantive amount of research seeking to investigate how texting and mobile instant messaging affect interpersonal relationships has used self-reported behaviors (e.g., Baber, 2012; Bradnam, 2017; Hu et al., 2004; Igarashi et al., 2005a, 2005b).

However, several studies have also investigated observed communication behavior. For example, Underwood et al. (2012) distributed Blackberry phones with prepaid plans to 175 students and collected all text messages, emails, and instant messages sent through these devices. From their results, they determined that self-reported use and actual use of the phone were not significantly correlated, highlighting the value of collecting behavioral measures in addition to self-reports (see also Parry et al., 2021, for a systematic review). Furthermore, they found no differences in texting behaviors by gender but were able to gain unique insight into the frequency of using sexual and profane language among teenagers by analyzing more than 40,000 text messages. These behaviors are difficult to assess through self-reports due to social desirability and experimenter effects (see above).

In a similar vein, Jensen and Hussong (2021) used over 500,000 donated text messages from college students to assess how they communicated about alcohol consumption. Using a dictionary-based approach, they found that their measure of “alcohol-talk” correlated with self-reported measures of alcohol consumption and related risk behaviors and underlined how this scalable measure could be used to assess norms around alcohol consumption in peer networks.

In another recent study, Brinberg and Ram (2021) used more than 1 million text messages donated by 41 romantic couples from their iMessage chat histories to investigate whether couples linguistically aligned with each other over time. Using three different measures, they found support for the hypothesis that couples’ language becomes more similar over time as their relationship matures, until reaching an optimal plateau. Using the same dataset, Brinberg et al. (2021) also investigated how texting behaviors change when couples transition into romantic relationships. They found that daily frequency of texting but not responsivity to messages or daily message length followed an inverted U-function that peaked around the time of becoming a couple, providing valuable insights for theoretical constructs such as relational trajectories (e.g., Eastwick et al., 2018; Surra, 1985, as cited in Brinberg et al., 2021) and relational transition models (e.g., Solomon et al., 2016, as cited in Brinberg et al., 2021).

In a broader study about the digital socializing behaviors of young adults, Harrari et al. (2020) collected call and text messaging behaviors, app usage inferred from phone logs, and conversation behaviors inferred from the phones’ microphone in conjunction with a personality questionnaire. For this study, the authors used four different samples with almost 1000 respondents in total. Even though measures differed between samples, and not all measures were collected in all samples, the authors found that sociable behaviors had high interpersonal variance and intrapersonal temporal stability, and correlated positively with each other. Furthermore, the study found correlations between sociable behaviors and the personality traits extraversion, openness, and neuroticism, underlining how objectively measured behaviors might provide insight into underlying interindividual differences on a psychological level (see also Marengo et al., 2023, for a recent review).

These examples highlight the value of using unobtrusive behavioral measures of interpersonal interactions to quantify interpersonal communication. However, they also highlight the limitations of using texting or instant messenger data, as studies are often restricted to specific hardware or operating systems, and require cooperation with phone companies or furnishing expensive smartphones to study participants. In the following paragraphs, we will thus provide an overview for a novel tool to facilitate the collection of WhatsApp chat logs.

## WhatsApp chat log data

WhatsApp is currently one of the most interesting sources of instant messenger data for social scientists, due to its vast user base, global popularity, diverse features, and heavy use among most users (Kemp, 2020; Kohne et al., 2022; Montag et al., 2015). Moreover, WhatsApp offers a convenient feature that permits users to export their chat logs as an unencrypted .txt file, making it relatively effortless to acquire data donations from potential research participants.^1^ These preconditions make WhatsApp data an intriguing candidate for research using data donations. In the following section, we will thus discuss the unique advantages and challenges for using donated WhatsApp chat log data for research about interpersonal interactions.

First, WhatsApp chat logs can quantify interpersonal communication in high granularity without subjectivity and memory biases within a given conversation (Kohne et al., 2022). People do not need to remember how often they talked to a person and for how long, because WhatsApp keeps a record of messages (the chat log) that are sent in each chat if they are not manually deleted or scheduled to self-delete. Moreover, this log prevents people from intentionally or unintentionally misreporting previous behaviors and putting themselves in a more favorable light. The chat logs therefore may deliver a more objective and valid source of data on interpersonal communication patterns than self-report data.

Second, exportable chat logs are a standard feature of WhatsApp and do not require much extra effort on the part of the participants. Potential participants do not have to carry additional devices or install any third-party software on their phones to export their WhatsApp chat log for a given conversation. The logs are thus more convenient for participants and likely scale better than most experiments, offline observational studies, and the use of many physiological measures.

Most importantly, though, the chat logs are obtained retrospectively, so that participants are not aware that their communication behavior will be investigated at the time the data are created (Kohne et al., 2022). In this respect, chat logs are similar to observational and archival studies, and provide researchers with data that are not biased by reactivity or experimenter effects, as opposed to self-reports, experimental studies, or the use of physiological measures.

However, WhatsApp chat logs also have several challenges and disadvantages. First, while chat logs provide insight into a specific online interaction channel, interactions through other online or offline channels remain uncovered. For example, for a cohabiting couple, most of their interactions will take place face-to-face, and their WhatsApp chat log might only be a sporadic exchange of grocery lists. Likewise, when other messenger apps such as Instagram messenger or Telegram are primarily used for online communication, looking at a WhatsApp chat log in isolation might result in a biased view of the nature of online interactions in a social relationship. A key disadvantage of WhatsApp chat logs for investigating interpersonal interactions and communication is thus the lack of contextual information about the social relationship and other communication channels in question.

Likewise, analyzing chat logs allows researchers to quantify interpersonal interactions in high resolution, but lacks the introspective aspect of self-reports. Basically, chat logs can tell us what happens in high detail and validity, but not necessarily why it is happening. For example, we might be able to pinpoint the exact time that somebody stopped replying in a WhatsApp chat, but that does not tell us w*hy* they stopped replying.

Finally, and most important for this paper, collecting and processing WhatsApp chat log data is a massive logistical challenge for scientists, because they require a custom infrastructure to be collected, processed, and stored in a secure, anonymous, ethically reflected, and General Data Protection Regulation (GDPR)-compliant manner. Previous studies that have used donated WhatsApp chat logs (Ueberwasser & Stark, 2017; Verheijen & Stoop, 2016) typically do not publicize the tools and infrastructure they used for data collection, so that the practical hurdles for collecting donated WhatsApp chat logs are usually prohibitively high for most research projects.

In sum, WhatsApp chat log data combine the advantages of reduced memory bias, reactivity, and subjectivity of observational and archival studies, with the advantages of scalability and ease of feasibility for participants that self-report measures provide. Moreover, they can allow a much more fine-grained view into everyday social interactions and communication patterns than most self-reports. However, they often lack contextual information about the social relationship and other channels of communication if those are not quantified otherwise, for example with an additional survey. In addition, the practical hurdles of collecting, processing, and analyzing donated WhatsApp chat logs are so steep that most researchers cannot work effectively with these data. To leverage the full scientific potential of donated WhatsApp chat logs, researchers would thus need a framework that allows them to combine other data collection paradigms (e.g., surveys) with transparently donated and effectively preprocessed WhatsApp chat logs. With the ChatDashboard framework introduced in the following section, we seek to provide a basis for that.

## ChatDashboard framework

The goal of the ChatDashboard framework is to make donated WhatsApp chat logs as accessible as possible for ethically reflected social science research. To achieve this goal, the process of collecting, processing, anonymizing, linking, and analyzing donated WhatsApp chat logs must be as easy and convenient as possible for participants and researchers alike, while still being transparent and secure, and giving participants as much control over their data as possible (see also Ohme & Araujo, 2022). In addition, the donated data should be processed in such a way that they are easily usable for social scientists using their regular tools and software for data processing and analysis.

**Fig. 1:**
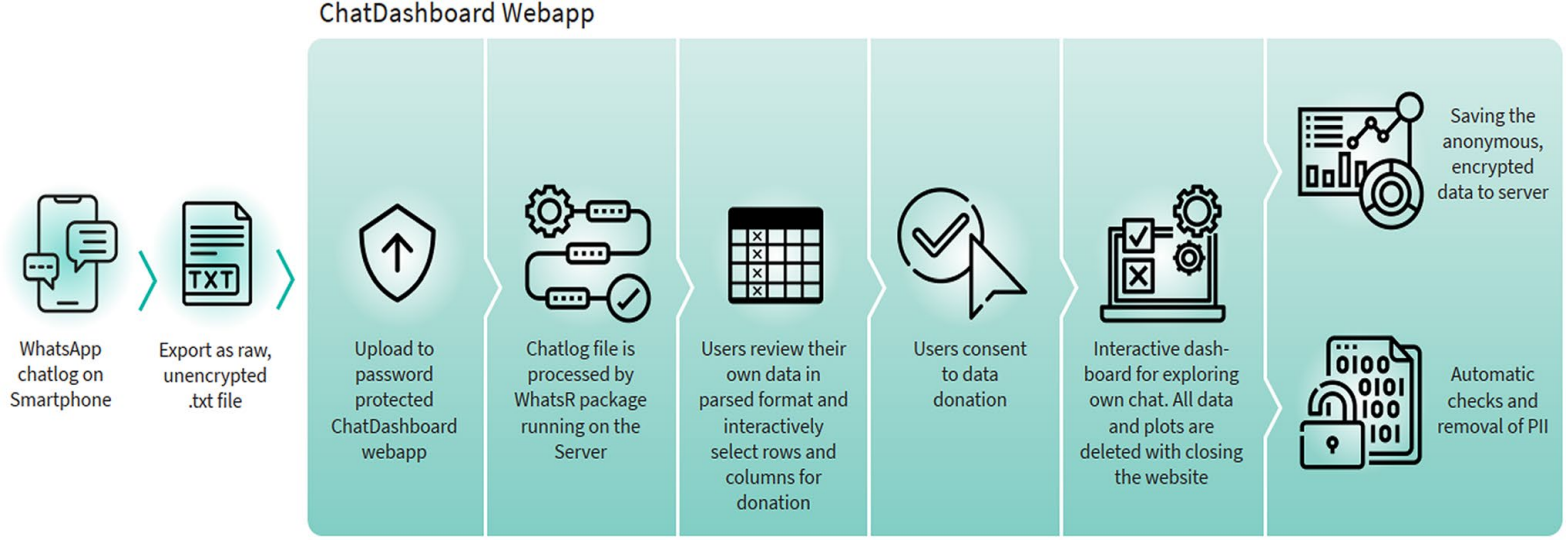
An overview of the data donation process with ChatDashboard framework. Personal identifiable information (PII) is removed automatically, and only self-selected data are encrypted and saved on the host server. Icons are from https://www.flaticon.com/

To achieve these goals, we decided to develop ChatDashboard as a modular framework consisting of three parts. These modules are the WhatsR R package^2^ as a backend for parsing, preprocessing, anonymizing, checking consent for, and visualizing donated WhatsApp chat logs; the ChatDashboard R Shiny web app^3^ for uploading, reviewing, and securely donating WhatsApp chat logs; and DashboardTester,^4^ an automated script for testing the correct setup of the framework by simulating participants. All components were developed in R to maximize customizability and adaptability for social scientists even without extensive programming knowledge. All necessary dependency packages are freely available, as is the source code of the WhatsR R package, the ChatDashboard app, and DashboardTester. All components come with the GPL3 license^5^ and can be freely used and adapted for research with proper attribution in derivative works. In the following sections, we will discuss each of the modules in greater detail and explain the decisions that shaped their design (see also Fig. 1).

## The WhatsR package

The WhatsR R package is the most fundamental part of the ChatDashboard framework. It is designed to enable researchers to transform exported, unstructured WhatsApp chat logs from .txt format into well-structured, rectangular data frames with one row per sent message and one column for each feature extracted from the message. Furthermore, it allows researchers to anonymize chat logs, exclude non-consenting chat participants, calculate basic statistics, and create visualizations of chat logs. While other R packages (e.g., Gruber, 2023) exist for parsing extracted chat logs, this wide range of features for enabling scientific data collection is so far unique to the best of our knowledge. As such, the WhatsR package is agnostic to how WhatsApp chat logs were collected and can be used in different data collection paradigms, from donated chat logs (Seufert et al., 2015; Ueberwasser & Stark, 2017; Verheijen & Stoop, 2016) to researchers joining public chat groups (Garimella & Tyson, 2018; Machado et al., 2019; Melo et al., 2019; Narayanan et al., 2019; Resende et al., 2019), or to artificially created chats in experiments (Sprugnoli et al., 2018). In total, the WhatsR package contains 20 functions that can be grouped into functions for processing chat logs, visualizing results, computing summary statistics, keeping the package up to date, and simulating artificial chat logs for testing purposes (see Table 2). In addition, the package contains supplementary files to enable text processing, such as lists of regular expressions (regex)^6^ for operating system (OS) and language detection, separating chat content generated by users from chat content inserted by WhatsApp, and for detecting smileys and emojis.

Importantly, though, WhatsApp chats displayed on a person’s phone might contain several features that are not represented in the exported chat logs. These features include reactions to individual messages using emojis, quoting individual messages to directly reply to them, live voice and video calls, and all sent media content such as images, audio, video, or documents. In the case of emoji reactions or quoting messages, there is no indicator at all in the exported chat logs, so the WhatsR package unfortunately cannot detect these features. For the sent media content, it depends on how the chat log is exported by a participant. Using the “Include media” option, participants are sent a chat log with up to 10,000 messages and the media files as separate files. The chat log will contain the file names as the message content when a file was sent. In contrast, when using the “without media” option, participants are sent only a chat log with up to 40,000 messages and no additional media files. The chat log will also not contain file names, but only an OS-specific indicator that a media file was sent, which is detected by WhatsR and listed in a separate variable (see Table 1). For live voice and video calls, there is an indicator in the chat log showing that a call took place, but no further information about the duration or contents of these calls is available. The WhatsR package can thus only detect that these calls took place, and saves the corresponding indicator in the system messages column (see Table 1).

**Table 1:** Overview of columns in data frames parsed by the parse_chat() function and their respective anonymized and non-anonymized versions. Using parse_chat() with anonymize = FALSE will return a data frame with the 15 columns listed below. Using anonymize = TRUE will return a data frame containing 11 columns and will use anonymized versions for the columns Sender, URL, Media, and Location. Using anonymize = “add” will return a data frame with 19 columns, in both raw and anonymized form.

Column Name	Description	PII	Anonymization
DateTime	Timestamp for date and time the message was sent. Formatted as yyyy-mm-dd hh:mm:ss	no	none
Sender	Name of the sender of the message as saved in the contact list of the exporting phone or telephone number. Messages inserted by WhatsApp into the chat are coded with “WhatsApp System Message”	yes	PII replaced with placeholders
Message	Text of user-generated messages with all information contained in the exported chat log	yes	deleted
Flat	Simplified version of the message with emojis, numbers, punctuation, and URLs removed. Better suited for some text mining or machine learning tasks	yes	deleted
TokVec	Tokenized version of the Flat column. Instead of one text string, each cell contains a list of individual words. Better suited for some text mining or machine learning tasks	yes	deleted
URL	A list of all URLs or domains contained in the message body	yes	URLs shortened to domains
Media	A list of all media attachment filenames contained in the message body	yes	Filenames shortened to file extensions
Location	A list of all shared location URLs or indicators in the message body, or indicators for shared live locations	yes	Coordinates are replaced by indicator
Emoji	A list of all emoji glyphs contained in the message body	no	none
EmojiDescriptions	A list of all emojis as textual representations contained in the message body	no	none
Smilies	A list of all smileys contained in the message body	no	none
SystemMessage	Messages that are inserted by WhatsApp into the conversation and not generated by users	yes	deleted
TokCount	Amount of user-generated tokens per message	no	none
TimeOrder	Order of messages as per the timestamps on the exporting phone	no	none
DisplayOrder	Order of messages as they appear in the exported chat log	no	none

**Fig. 2:**
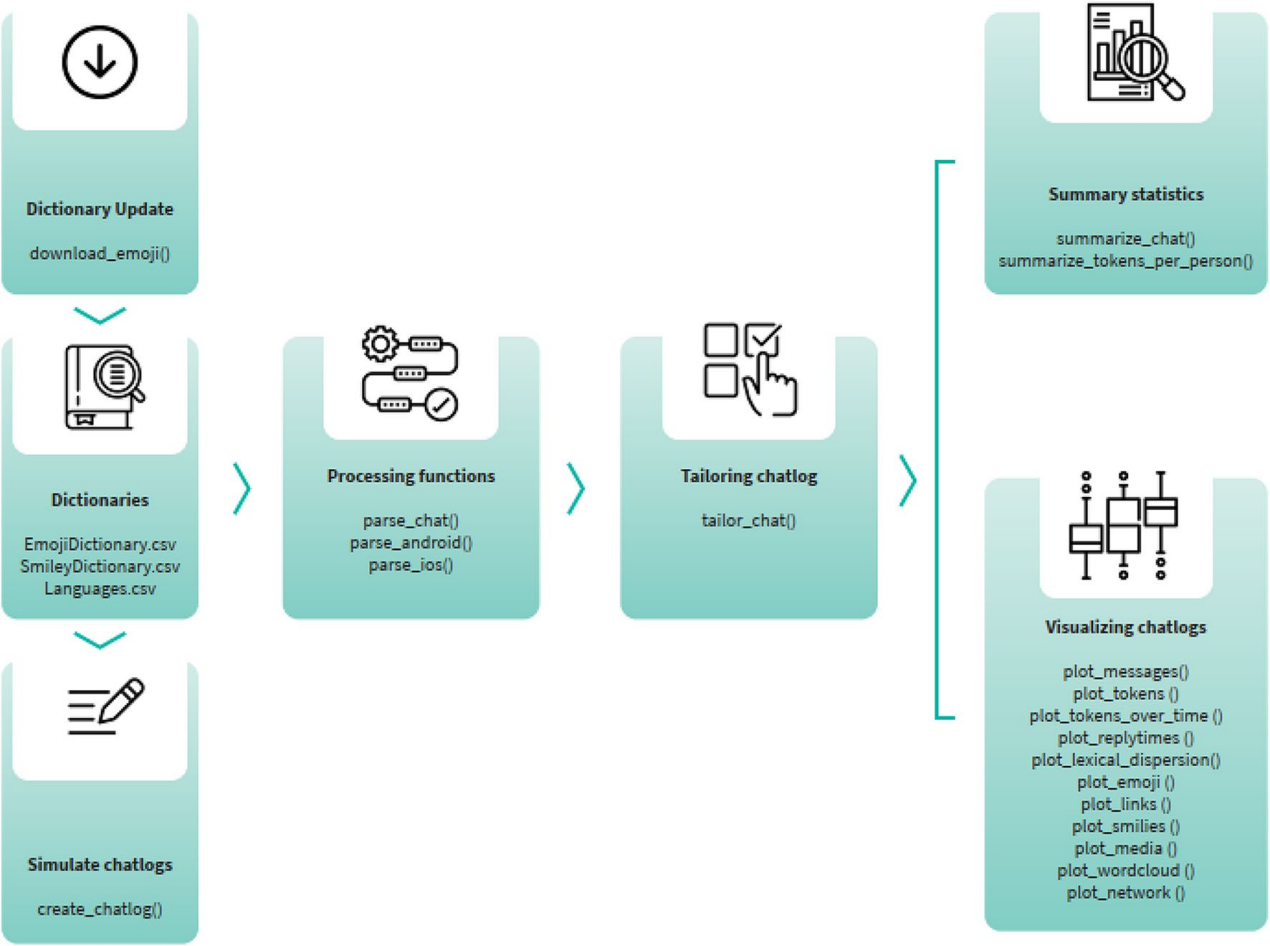
Overview of the functionalities of different functions and a typical workflow for processing a raw WhatsApp chat log using the WhatsR package. Icons are from https://www.flaticon.com/

In the following sections, we will describe the functions and features of the WhatsR package in greater detail (for an overview, see also Fig. 2 and Tables 1 and 2), and describe how they can be used to preprocess WhatsApp chat logs. For an example of the raw structure of donated WhatsApp chat logs, see Kohne et al. (2022).

**Table 2:** Overview of all functions contained in the WhatsR package and their respective features

Type	Name	Features
Processing functions	parse_chat()	Takes an exported WhatsApp chat log as input and converts it into a data frame with one row per message and 15 feature columns (see Table 1). It has additional parameters for anonymizing the chat log and for removing messages from non-consenting participants
	parse_ios()	Subfunction of parse_chat(). Separates the raw text file into different messages and distinguishes user-generated messages from WhatsApp System messages as per the iOS chat log structure
	parse_android()	Subfunction of parse_chat(). Separates the raw text file into different messages and distinguishes user-generated messages from WhatsApp System messages as per the Android chat log structure
Visualization functions	plot_messages()^a^	Function for visualizing the number of messages per sender as a bar plot, cumulative sum, heatmap, or pie chart
	plot_tokens()^a^	Function for visualizing the distributions of tokens sent per sender and message as a bar plot, box plot, violin plot, or heatmap
	plot_tokens_over_time()^a^	Function for visualizing the number of tokens per sender across time. Includes visualizations per year, per month, per day, per hour of day, per day of week, and all time
	plot_smilies()^a^	Function for visualizing the number of smileys sent per sender as a bar plot, cumulative sum, heatmap, or split bar plot
	plot_emoji()^a^	Function for visualizing the number of emojis sent per sender as a bar plot, cumulative sum, heatmap, or split bar plot
	plot_links()^a^	Function for visualizing the number of links or domains sent per sender as a bar plot, cumulative sum, heatmap, or split bar plot
	plot_media()^a^	Function for visualizing the number of media files or file types sent per sender as a bar plot, cumulative sum, heatmap, or split bar plot
	plot_wordcloud()^a^	Function for visualizing word clouds from tokenized versions of messages. Essentially a wrapper for the ggwordcloud R package^b^
	plot_network()^a^	Function for visualizing networks of user interactions in WhatsApp chat logs. Essentially a wrapper to the visNetwork R package.^c^ Constructs an edge between two users for each consecutive message. Edges can be built based on sent tokens, emojis, smileys, locations, URLs, media files, or amount of sent messages
	plot_lexical_dispersion()^a^	Function for visualizing occurrence of specific tokens in the sent chat messages. Requires raw message texts to be present in the data frame
	plot_replytimes()^a^	Function for visualizing the distribution of time delay for responding to a previous message, or being responded to, for each participant in the chat
	plot_locations()^a^	Function for visualizing sent locations from within the chats on a map. Essentially a wrapper for the ggmap R package.^d^ Requires non-anonymized chat logs as input. Temporarily not available in CRAN version due to pending changes in a dependency package.
Summary functions	summarize_chat()^a^	Function for summarizing basic statistics about a WhatsApp chat log. Contains number of messages, tokens, participants, system messages, emoji, smileys, links, media files, and locations. Also computes datetime of first and last message and total duration of the chat
	summarize_tokens_per_person()^a^	Function for summarizing basic statistics about tokens sent per person. Contains timestamp of first and last message and distribution of sent tokens for each chat participant
Helper functions	download_emoji()^a^	Helper function for scraping a dictionary of emojis from the Unicode website^e^ and building a corresponding data frame. Can be used to update the built-in emoji dictionary manually if new emojis are added to WhatsApp
	tailor_chat()^a^	Helper function to restrict a parsed WhatsApp chat log to specific timeframes or senders, or to exclude WhatsApp system messages
Testing function	create_chatlog()^a^	Function for creating files with the same structure as exported, unparsed WhatsApp chat logs using artificial names, telephone numbers, and lorem ipsum message text.^f^ Contains parameters to control the operating system, language settings, time settings, first and last timestamp of the message, and number of users, emojis, unique emojis, links, locations, smileys, unique smileys, media, and self-deleting photos in the chat. These files can be used for testing the correct setup of the ChatDashboard framework (see section 3)

^a^All visualization functions have parameters for restricting plots to specified timeframes and senders, and for excluding system messages from plots. They return either a customizable ggplot2 object or the preprocessed data frame.

^b^https://cran.r-project.org/web/packages/ggwordcloud/vignettes/ggwordcloud.html

^c^https://www.rdocumentation.org/packages/visNetwork/versions/2.1.2

^d^https://cran.r-project.org/web/packages/ggmap/readme/README.html

^e^https://unicode.org/Public/emoji/15.1/emoji-test.txt

^f^https://en.wikipedia.org/wiki/Lorem_ipsum

### Parsing WhatsApp chat logs

The parse_chat() function in the WhatsR R package takes a raw, unencrypted WhatsApp chat log as input and returns a rectangular R data frame with one row per message in the chat and several columns with features extracted from the messages. This is no trivial task, as the structure of the exported chat logs depends on multiple factors including the OS of the exporting phone, its language settings, and the date and time settings of the phone. To achieve this, parse_chat() uses regexes to automatically detect the language setting, the date and time settings, and the OS of the exporting phone from the first 10,000 characters of the exported chat log.

To detect the OS of the exporting phone, the parse_chat() function uses the fact that timestamps are formatted differently for WhatsApp chat logs exported from iOS and Android phones (see Kohne et al., 2022, for an example), and counts all occurrences of OS-specific timestamps in the chat log. Afterwards, the function automatically assumes the OS of the exporting phone to be the one with the most corresponding timestamps in the chat. If the system cannot automatically assume the OS with this method, researchers are prompted to indicate it manually.

The language detection in parse_chat() is implemented by comparing the language of system messages in the chat log to a list of corresponding regular expressions that come with the package in a file called “Languages.csv”. System messages are messages inserted into chat logs by WhatsApp to comment on certain user actions (e.g., “Messages and calls are end-to-end encrypted. No one outside of this chat, not even WhatsApp, can read or listen to them. Tap to learn more.”, “You have changed the groups profile picture”, etc.). Because these system messages are not generated by participants, they accurately reflect the language settings of the exporting phone, independently of the language that is used by the participants in the chat. For example, if a participant had their phone settings in German but donated a chat where they conversed with a friend in English, the system messages of the chat log would still be in German. The parse_chat() function would then count the occurrences of German and English system messages in the chat log, and correctly assume the language setting to be German. Currently, only English and German are supported by parse_chat(), but the package can be extended to support other languages as well. To do so, the “Languages.csv” file in the package can be appended with regular expressions to detect WhatsApp system messages in the respective language, and several lines of code in the parse_chat() function would have to be adapted.

After determining the correct settings for the language and OS of the exporting phone, parse_chat() removes all left-to-right markers^7^ and zero-width no-break spaces^8^ from the chat logs. These invisible control sequences are used to control the display of text in applications, but might distort natural language processing in R. Afterwards, the chat log is passed over to one of two helper functions, parse_andorid() or parse_ios(), to further process the chat log according to its OS-specific file structure.

In these functions, trailing line breaks are replaced by a text indicator (“ start_newline ” by default), as are OS-specific indicators for omitted media files (“ media_omitted ” by default)^9^ to facilitate natural language processing later. Subsequently, parse_ios() and parse_android() use the respective OS-specific regular expressions to split the chat log into a list of individual messages by cutting the chat log in front of the timestamp that precedes every message. The messages are then further split into three parts each: the timestamp, name of the sender, and the body of the message. The timestamps are extracted from the messages using regex, and formatted as class POSIXct^10^ using the parse_date_time() function from the lubridate package (Grolemund & Wickham, 2011). This conversion allows researchers to compute time differences between messages and use the timestamps accurately for visualizing variables across time. The names of senders of messages are extracted and converted into a factor variable. Importantly, the senders are represented exactly as in the address book of the exporting person’s phone and can be either names or telephone numbers if the sender is not saved in the exporting person’s address book (Kohne et al., 2022). Formatting the sender’s name as a factor allows their use as a grouping variable in ggplot2 visualizations, and to compute aggregated statistics using tidyverse functions (Wickham et al., 2019). Message bodies are provisionally kept as a regular character string variable in this step. From these message bodies, the parse_android() and parse_ios() functions also extract sent locations (represented by Google Maps links with latitude and longitude^11^) and indicators for sent live locations (indicated by the message string “live location shared”) into separate variables. The chat logs are thus turned into data frame objects with five variables (timestamp, sender name, message body, sent media files, and sent locations) and one row per message, and handed back to the parse_chat() function for further processing.

Because some messages are WhatsApp system messages and do not contain a sender name, their messages bodies are erroneously parsed into the sender column at first. In a next step, these falsely parsed sender names are corrected by replacing them with an indicator for WhatsApp system messages (“WhatsApp System Message” by default). This makes it possible to differentiate between message content that was produced by participants and message content that was inserted into the chat by WhatsApp. The identification of system messages is implemented by matching them to an OS- and language-specific, predefined list of regular expressions (see “Languages.csv”) for all system messages. Currently, this list only contains regular expressions for English and German system messages, but it can be expanded in the future (see above).

After differentiating the system messages from user-generated content in the sender column, the parse_chat() function extracts additional features from the message bodies. First, the rm_url() function from the qdapRegex package (Rinker, 2022) is used to extract all contained links into a separate list. Likewise, all contained emojis are extracted from the message bodies into a separate list using regex and a custom-built dictionary that is part of the package (“EmojiDictionary.csv”). Different dictionaries can be used with the “emoji_dictionary” parameter in parse_chat(). Importantly, we implemented the extraction of emojis based on a procedure from the rwhatsapp package (Gruber, 2023), because it proved to be considerably faster than the default string matching methods in the stringr (Wickham, 2022) and stringi R packages (Gagolewski, 2022), or the rm_default() and mgsub() functions from the qdapRegex and mgsub packages, respectively (Ewing, 2021; Rinker, 2022). We add the emojis as two different columns to the parsed data frame, once as glyphs (e.g., “  ”) and once as textual descriptions (e.g., “Smiling_Face_with_Smiling_Eyes”). Similarly, we use either the ex_emoticon() function from the qdapRegex package or a custom-built dictionary (“Smilies.csv”) that is also contained in the package^12^ to extract all ASCII smileys (e.g., “ :-) ”) from the message bodies into a separate list. Researchers can specify which option they prefer by using the “smilie_dictionary” parameter in the parse_chat() function.

For many natural language processing and text mining packages in R, text data need to be preprocessed so that they do not contain links, smileys, emojis, mentions, punctuation, capitalization, line breaks, or numbers that would distort the analysis in question (e.g., Ignatow & Mihalcea, 2016; Wiedemann, 2016). To facilitate this step, the parse_chat() function also preprocesses the message bodies into two additional, separate columns that only contain words sent by users, either as a whole text string (in a column called “Flat” for the plain text messages) or as a list of individual words (in a column called “TokVec” for token vector). To create these columns, we start with the message bodies and first remove all WhatsApp system messages from the strings using regex. In the next step, all links and other non-word entities are removed from the messages using the rm_url() and rm_non_words() functions from the qdapRegex package (Rinker, 2022). This already gives us the “Flat” column, which is then split into a list of vectors of tokens per sent message, containing all individual tokens using the tokenize_words() function from the tokenizers package (Mullen et al., 2018). We thus, on the one hand, keep a human-readable message column (“Message”), that contains all elements that are also contained in the message displayed on the phone, but on the other hand, provide a machine-friendly version, containing only word tokens sent by users. This machine-friendly version is present in two forms, once as a whole text string (“Flat”), and once as a list of tokenized, lowercased words (“TokVec”). This enables researchers to do both qualitative and quantitative analyses with the final data frame and use the “Flat” and “TokVec” columns as input for natural language processing or machine learning models without the need for excessive additional preprocessing. Of note, though, we are not implementing any content-altering preprocessing steps such as removing stopwords, concatenating frequently collocated words, or stemming. We argue that these steps are best left to each individual researcher, because such steps should be a conscious, documented decision for each research project and not preset through the data collection infrastructure.

In the next step, all created variables are pasted together into one data frame with one row per message and 13 different variables (see Table 1). However, because some variables (specifically: TokVec, Emoji, URL, Emoji, EmojiDescriptions, and Smilies) are lists with potentially multiple values per message, they do not necessarily have the same length when unlisted as the other variables (DateTime, Sender, Message, Flat, Media, Location, and SystemMessage). By design, the data.frame() function in R does not allow multiple values per cell in a data frame object. However, to keep the parsed data frame as intuitive and easy to use as possible, we circumvent this issue by encasing the list variables with the I() function, essentially forcing data.frame() to accept multiple values per cell (R Core Team, 2022). To this data frame we also add the number of user-created words per message by simply counting the number of tokens in the “TokVec” variable and adding them as a 14th, numeric variable to the data frame (“TokCount”). In a final step, two more numeric variables are added to display the ordering of messages in the chat log. Importantly, it can happen that the order of messages as they are extracted from one phone is different from the order of messages on another phone. From our personal experience, we suspect that this happens when there is no internet connection on at least one of the phones while multiple people in the conversation try to send messages simultaneously. To transparently check for and deal with this potential issue, we include two indicator variables for the order of messages in the chat log (“DisplayOrder” and “TimeOrder”). One variable indicates the order of the messages as they were exported from the phone of the data donator, while the other indicates the order according to the timestamps of the messages in the exported chat log. In nearly all cases, these two variables will be identical, but we still include them to enable sanity checks.

The algorithm for the parse_chat() function described above results in a parsed data frame with one row per sent WhatsApp chat message and 15 variables containing different features of the messages (see Table 1). The data frame keeps both a human-readable and a machine-interpretable representation of the message contents and thus enables qualitative and quantitative analyses of chat log data. It can be readily used to calculate statistics or create visualizations, or as a basis for advanced NLP or machine learning models. However, two issues inherent to donated WhatsApp chat logs still remain: Many columns still contain personal identifiable information (PII) such as names, telephone numbers, or all kinds of information that people might chat about, and the consent of research participants for their data being processed needs to be validated externally. The parse_chat() function offers parameters to deal with both of these issues. While an in-depth discussion of these issues from a methodological perspective can be found elsewhere (Kohne et al., 2022), we will provide an overview of addressing these issues from a technical perspective in the following sections.

### Anonymizing WhatsApp chat logs

Depending on the research question at hand, researchers might need access to raw message data (García-Gómez, 2018; Sampietro, 2019; Sprugnoli et al., 2018). In other cases, however, anonymous user data are sufficient to answer the respective questions (Narayanan et al., 2019; Seufert et al., 2015, 2016), and researchers should be as parsimonious as possible with using PII (see also Kohne et al., 2022) . For this reason, the parse_chat() function contains a parameter to anonymize donated chat log data, if desired, while still enabling the differentiation between different senders. Starting from the parsed data frame described above, columns that potentially contain PII are the “Sender” column, the “Message,” “Flat,” and “TokVec” columns, the “URL” column (e.g., links to social media profiles), the “Media” column (e.g., file names containing PII), the “Location” column, and the “SystemMessage” column (group names, participant names, or telephone numbers). These columns must either be deleted or anonymized to ensure that no PII is present in the anonymized version of a chat log.

To implement this, setting the “anonymize” parameter to “TRUE” in the parse_chat() function, deletes the “Message,” ”Flat,” “TokVec,” and “SystemMessage” columns from the returned data frame. As has been outlined elsewhere (Finck & Pallas, 2020; Moretón & Jaramillo, 2021; Mozes & Kleinberg, 2021, as cited in Kohne et al., 2022), it is extremely hard to truly anonymize textual data with certainty because we can never know what exactly is contained in the messages, and in which formats. Even though there are some automated methods to anonymize text data (e.g., Kleinberg et al., 2022), they usually rely on predefined assumptions about what kind of personal data can be contained in the input and in which formats. Because such methods might break down when people make spelling mistakes, use slang or dialect, mix languages, or use ASCII art or smileys to express PII, we decided to completely remove these columns. Similarly, the sent locations are replaced with placeholders, as location data are notoriously difficult to anonymize as well (Gambs et al., 2014; Zang & Bolot, 2011).

In contrast to this anonymization by removal, other instances of PII can be reliably anonymized without removal. In the sender column, usernames and telephone numbers are replaced by placeholders in such a way that no PII remains in the column, but it is still discernable which messages were sent by the same senders (e.g., Person_1, Person_2, etc.). To achieve this, we replace the factor levels of the “Sender” column (representing all unique senders) with a consecutively numbered placeholder. Only WhatsApp system messages are exempt from this so that these can still be identified even in the anonymized chat logs. Similarly, for the media column, file names are removed using regex but the file type extensions are retained. This allows researchers to still see which (anonymous) persons send which types of files how often, but removes the potential for PII being contained in the file names (e.g., “John_Smith_Birthday_Invitation.jpg” is reduced to just “.jpg”). Importantly, media file names are only present in the raw exported chat logs in the first place if they are exported using the “Include media” option.^13^ When selecting the “without media” option, only .vcf file names (attached contacts) are included in the chat log (Kohne et al., 2022). For links extracted from the messages, the full link is reduced to the subdomain and domain, but all paths, queries, parameters, or fragments are removed (e.g., “https://twitter.com/JuuuuKoooo” would be reduced to just “https://twitter.com/”). All other variables that do not potentially contain PII are neither removed nor altered (see Table 1).

In sum, parsing chats with the anonymize parameter set to TRUE results in a data frame with 11 columns that do not contain PII but still keep as much data as possible usable, and potentially shareable for reproducibility (see Table 1). The “anonymize” parameter can also be set to FALSE, which will keep all PII in the parsed chat logs, and to “add” which will keep both the raw and the anonymized versions of the columns in the parsed chat logs.

### Checking opt-in consent in WhatsApp chat logs

Another frequent issue when working with donated WhatsApp chat log data is the question of how to ensure that informed consent is obtained from chat participants. So far, different researchers have approached this issue differently, from not obtaining consent to obtaining consent from one of the chat participants, to asking one chat participant to ensure that all participants consent (see Kohne et al., 2022, for an overview). While the question of which method for obtaining consent is appropriate depends on a variety of factors, including the types of data, the mode of processing, and whether the data are shared afterwards, so far there seems to be no ready-to-use technical solution to ensure that only messages from consenting chat participants are donated. The parse_chat() function provides the basis for such a system with the “consent” parameter. While being NA by default, any string passed via this parameter will automatically check all messages for the presence of the string and delete all messages from participants that did not post the exact string as a message into the chat. In essence, this function can be used to instruct participants to ask their chat partners to post a specific consent message into the chat before extracting and donating the chat log. Consequently, the data produced by all participants of the chat who did not post the consent message into the chat will be automatically removed by parse_chat(). The parameter therefore serves as a low-threshold solution to automatically check for opt-in consent of all chat participants.

In sum, the parse_chat() function provides a foundation for researchers to parse and preprocess donated WhatsApp chat logs, extract variables of interest from them in both human-readable and machine-friendly formats, restrict and anonymize donated chat logs, and automatically delete messages from chat participants who did not provide active opt-in consent. In addition to these features, the WhatsR package provides other functions (see Table 2) to help researchers tailor parsed chat logs, compute basic statistics, create visualizations for parsed chat logs, keep the internal emoji dictionary up to date, and create artificial chat logs for testing purposes. By design, these artificially created chat logs do not contain any personal information but still have the same structure as real chat logs. They are useful for testing WhatsApp data donation pipelines before participants can donate real chat logs (see section “The DashboardTester script” for details). In the following section, we will briefly outline the visualization and helper functions in the WhatsR package.

### Visualizing WhatsApp chat log files

The visualization functions in the WhatsR package are designed to help researchers to initially explore WhatsApp chat logs graphically and to facilitate further in-depth analysis. As such, they all take a data frame object parsed by parse_chat() as input and return either a ggplot2 (Wickham, 2016) object or the data frame used to create the visualization as a basis for custom visualizations. Which one of the outputs is returned can be controlled through the “return_data” parameter in all visualization functions. Furthermore, all visualization functions have parameters to restrict the input data to specific time spans, senders, or variables of interest to be visualized (see Table 2). Some of these functions only work with data frames still containing the message content (e.g., plot_wordcloud(), plot_lexical_dispersion()), while others also work with anonymized versions of the chat log. Importantly, the visualization functions can also be used to offer research participants feedback about their own communication behavior, which can be used as an incentive to donate data in the first place (cf. Schwind & Seufert, 2018). Some examples of visualizations can be found in Appendix Figs. 3, 4 and 5.

### WhatsR helper functions

The WhatsR package also contains two helper functions. One of these functions, download_emoji(), is used to download an updated version of an emoji dictionary from the Unicode website,^14^ should new emojis be added in the future. This can be used either to build an updated version of the WhatsR package or to create a custom emoji dictionary to be used in parse_chat(). The other helper function, tailor_chat(), can be used to restrict parsed chat logs to specific time spans or senders. This enables researchers to manually exclude chat participants, for example based on participation rates in group chats, or to restrict chats to specific times, for example to the duration of COVID-19 lockdowns or other specific events.

### Simulating artificial chat logs

Lastly, the WhatsR package also contains a function for creating artificial chat logs with the same structure as exported, unparsed WhatsApp chat logs with artificial names, telephone numbers, and lorem ipsum message text.^15^ The function offers parameters to control the OS, language settings, time settings, first and last timestamp of the messages, number of users, emojis, unique emojis, links, locations, smileys, unique smileys, media, and self-deleting photos in the generated chat logs. In essence, the function can be used to simulate chat logs with different phone settings and chat participant constellations or behaviors. These can be used for testing the WhatsR functions (and also whole data donation pipelines, see section “The DashboardTester script”) without using chats that could contain PII and are difficult to obtain consent for.

### Summary

In sum, the WhatsR package makes donated WhatsApp chat logs more accessible for social science researchers. It has functions to parse chat logs exported from iOS and Android phones, extract features of interest from the chat logs, and convert them into a rectangular R data frame with one row per message and one column per feature. This format allows for both qualitative and quantitative analyses of donated chat logs because it provides access to features in both human-readable and machine-friendly formats. The extracted features are formatted in a way to ensure that they only need minimal further processing to be usable in state-of-the-art visualizations, text mining, and statistical analysis packages in R. Furthermore, parse_chat() allows for automatic anonymization of chat logs and can exclude messages from participants who did not provide active opt-in consent.

These features make WhatsR a suitable basis for collecting data from research participants in an ethically reflected manner. On top of this, the package provides additional functions for tailoring and visualizing the parsed chat logs using a variety of plotting functions (see Table 2). These functions return either a customizable ggplot2 plot or the data frame used to create the plots, so that researchers can either use the built-in visualizations or easily create custom ones. Finally, the package also contains a function to simulate artificial chat logs with the same structure as real chats but with no PII for testing the functions of the WhatsR package, or even whole data donation pipelines built around the package (see section “The DashboardTester script”).

## The ChatDashboard web app

While the WhatsR package provides a basis for processing, anonymizing, consent-checking, and generating visualizations from WhatsApp chat logs, researchers still face the issue of how to collect WhatsApp chat logs in the first place. ChatDashboard is an open-source R Shiny web app that can be hosted by researchers themselves. The data donation platform enables them to effectively collect donated WhatsApp chat log data. The web app allows participants to upload their chat logs to a password-protected environment, transparently review and select parts of their chat logs, and donate them as an encrypted file. Furthermore, it can be used to give participants access to interactive visualizations into their own chatting behaviors, as an incentive for donating data (cf. Schwind & Seufert, 2018; Seufert et al., 2015). Using ChatDashboard can ensure that researchers only ever have access to reviewed, anonymized, donated, and encrypted data instead of having to interact with the raw, exported chat logs still containing PII. In addition, ChatDashboard can be configured to enable researchers to anonymously link donated WhatsApp chat logs to other sources of data, such as survey responses or other donated files. It thus enables researchers not only to collect WhatsApp data donations in a transparent way, but also to gather additional information to contextualize what is happening in the chat logs (see section “WhatsApp chat log data”). Most importantly, though, ChatDashboard is completely customizable, and researchers can adapt the web app as they see fit to collect anonymized data, raw data, or to add their own anonymization and preprocessing routines (see below).

ChatDashboard is built as an R Shiny web app^16^ and is thus comparatively easily accessible to social scientists looking to host their own WhatsApp data donation platforms. All necessary dependency packages are available open-source, as is the source code of the ChatDashboard app and the WhatsR package as a processing backend. In addition we provide a testing script for checking the correct setup of data donation pipelines, called DashboardTester (see section “The DashboardTester script”). All components come with the GPL3 license and can be freely used and adapted for research purposes with proper attribution. In the following section, we will describe in greater detail how ChatDashboard works and which decisions shaped its design. For a step-by-step guide on how to set up and customize ChatDashboard for yourself, please refer to our supplementary materials on our GitHub page.^17^ For showcasing the functionality of ChatDashboard, we have also set up an instance where no uploaded data are saved.^18^ You can try out the platform yourself by appending “/?id=” followed by a username of your choice to the URL and hitting “enter.” You are now able to log in with your selected username and “password” as a password.

The ChatDashboard web app comes as a collection of files with a script for running the app, a list of credentials for authenticating users with the app, a folder to contain used images and other files, and a folder for containing donated, parsed, and encrypted chat log files (see Table 3). The script for hosting the web app consists of two parts. One is for defining the user interface (UI) and everything that is displayed to the user (the environment is called “ui” in the script), while the other is for the server, defining all necessary actions and computations that need to be executed in the background (the environment is called “server” in the script). In the following section, we will provide a walkthrough from the perspective of a research participant to explain the functionality of ChatDashboard in detail.

**Table 3:** List of all files and folders necessary to run ChatDashboard on a server as a R Shiny web app

File/folder	Function	Relative path
ServerFolder	Folder containing the public RSA key file generated by the cyphr package for encrypting user data on the server	./ServerFolder
UserData	Folder containing the reviewed, encrypted chat log files	./UserData
www	General folder for additional files (e.g., images, logos, fonts) called from app.R to be included in the web app^a^	./www
app.R	Script containing all code for displaying frontend (“ui”) and handling processing in backend (“server”) of the ChatDashboard web app	./app.R
credentials.rds	R data frame object for storing valid user credentials if authentication should be handled with predefined IDs	./credentials.rds
favicon.ico	Icon to display to users as a logo for the web app on the left side of the address bar in their browser	./favicon.ico
README.md	Readme file for an overview of ChatDashboard and how to set it up	./README.md
LICENSE	Terms of the GPL3 license	./LICENSE

*Note*: For a step-by-step guide on how to set up ChatDashboard for yourself, please refer to our supplementary materials on our GitHub page. (See https://github.com/gesiscss/ChatDashboard/blob/master/README.md)

^a^See also https://shiny.rstudio.com/articles/tag-glossary.html

### Referral and authentication

Participants that are clicking on a link to the ChatDashboard web app first need to authenticate with a valid username and password to be able to access the platform (see Appendix Fig. 6). Authentication, on the one hand, ensures that only invited study participants can access the app and the server is not overloaded with too many uninvited users, and, on the other hand, provides a base layer of security because all code is hidden until users have authenticated. The authentication is implemented using the shinymanager R package (Thieurmel & Perrier, 2022), which allows users to be authenticated by storing valid credentials in an R data frame object that is saved on the server. In essence, it allows researchers to predefine a list of valid username and password combinations and hand them out to their study participants, for example after finishing a survey. However, sometimes a study design might require participants to create their own IDs, or participant codes may be interactively generated by survey software. As in these instances researchers do not know the IDs in advance, ChatDashboard also has the possibility of using strings passed as URL parameters as valid usernames in combination with a predefined password. In the default setup, ChatDashboard uses an ID parameter passed through an URL^19^ as a username that enables login when combined with the correct password. The password still has to be handed out to research participants through some other channel, for example, at the end of a survey in advance of the data donation. This enables researchers to also use participant-generated, or automatically generated, IDs as usernames for ChatDashboard. ChatDashboard can thus be used as a tool for standalone WhatsApp chat log donations, or to link donated chat logs with results from surveys conducted on other platforms.

### Overview of platform

After authentication, participants are forwarded to an overview page where the nature of the study, the requirements for participation, informed consent information, etc., can be displayed. This page should only be necessary in studies without any previous survey components, as this information should otherwise be provided in advance, prior to starting the survey. The text and layout of this page can be easily adapted by editing the page “Overview” in the “ui” part of app.R script in the ChatDashboard directory (see Table 3). For more information about adding and editing text and layout in R Shiny apps, you can refer to the R Shiny documentation.^20^

### Exporting, uploading, and parsing data

After clicking the “I agree” button on the overview page, participants are forwarded to a page for uploading their WhatsApp chat log data. On the right side of the page there are two images explaining in a step-by-step fashion how to export chat logs via email from a given chat for Android and iOS phones, with an additional referral to the official guide by WhatsApp.^21^ After participants have exported their chat logs to their device, they can use the sidebar menu on the left to select a file and upload it to the server. The file upload is implemented using the fileInput() function from the Shiny R package (Chang et al., 2022) and is configured by default to only accept .txt files. The maximum file size that can be uploaded is defined through an options() call at the start of the app.R script and is set to 50 Mb by default. After the upload is confirmed, the .txt file is automatically parsed on the server using the parse_chat() function from the WhatsR package (see section 4) and saved in a reactive object called “data.” Importantly, these parsed data still contain PII but are only accessible to the participants themselves, as R Shiny web apps create separate sessions for each user.^22^ Researchers can also add additional preprocessing steps or custom anonymization procedures by editing the “server” part of the app.R script in ChatDashboard. Depending on the changes made, though, the “server” and “ui” parts will have to be adapted and tested accordingly. We thus recommend using the default version of the framework if at all possible and executing all additional preprocessing or custom anonymization after data are collected.

### Data review, selection, and donation

In a next step, participants are forwarded to a page to explore their own data and select parts of it for donation (see Appendix Fig. 7). On the right side of the page, they see a table containing the parsed data frame that was created from their uploaded chat log (see also section “ChatDashboard framework”). The table is implemented using the datatable() function from the DT R package (Xie et al., 2022). It thus has a search function, can be ordered in ascending or descending order for each column, and is structured with a pagination system, displaying 10 messages per page by default. On the left side, there is a sidebar menu with a dropdown list where participants can interactively select which columns to display in the table. The guiding principle behind this page is that participants can see what they consent to donating, before they make a data donation. More specifically, what participants see in the displayed table always corresponds to their current selection for data donation.

For transparency, the columns that can potentially contain PII (see Table 1) are highlighted in gray in the online table and the dropdown menu. Participants can see these columns but will not be able to donate them in the default version of ChatDashboard, even if they chose to select them for donation (see below). Importantly, however, researchers can customize whether and which variables are automatically removed by editing the Colnames_exclude_pii variable in the app.R script of ChatDashboard. This will also automatically adapt the table displayed to participants so that they always see non-donatable columns as grayed out. ChatDashboard can thus also be used to collect raw chat messages from participants if researchers decide that this is necessary for their research question at hand.

In addition to excluding whole columns, participants can exclude individual rows by clicking on them in the table and then clicking on “exclude rows” in the sidebar menu. This action can also be undone by clicking on the “restore rows” button, which restores all previously excluded rows. In this way, participants can interactively select which rows and columns they would feel comfortable donating—while transparently seeing what their data look like, and which information could potentially be contained. For maximum transparency, participants can also download their own data selection or all of their uploaded data by using two respective buttons below the table. Importantly, researchers can retrospectively determine from a data donation whether and how much data were manually excluded by study participants. For the columns, comparing the default columns created in the parse_chat() function and the ones defined to be automatically excluded in the Colnames_exclude_pii variable in ChatDashboards app.R script with the ones present in the donation will give an indication of which columns were manually excluded. For the rows, the “DisplayOrder” and “TimeOrder” columns provide a consecutive numbering of rows. Any gaps in this numbering point to manually excluded rows.

To donate data, participants can click on the button “donate selection” on the bottom of the sidebar menu. This will prompt a popup asking for additional confirmation to prevent accidental contributions. Should participants have a column selected that could potentially contain PII (see Table 1), the script automatically removes it from the donated dataset and informs users about the removal with an automatic popup message.

### Hashing and encryption

The data frame containing only the data selected for donation, excluding selected columns that have been defined to be automatically removed, is then assigned a name consisting of the ID that was used as a username to log into the platform, the time of donation, and a SHA512 hash^23^ of the chat log. The hash is a unique value created from the contents of the data frame. This enables researchers to match each donated dataset to a participant in their survey results or other data donated via their ID, and check for exact duplicates using the hash values, for example when a participant donates the exact same chat twice. The hashing is implemented using the digest() function from the digest package (Lucas, 2022) and “sha512” as the “algo” parameter.

Afterwards, the data frame is encrypted using the encrypt_object() function from the cyphr R package (FitzJohn, 2022), using a predefined RSA (Rivest–Shamir–Adleman) key pair, which is stored on the server. Keys can be created in advance of deploying ChatDashboard by the researchers using the cyphr package.^24^ RSA is an asymmetric encryption that uses two different keys for encryption and decryption, meaning that the key pair stored on the server cannot be used to decrypt the data again. This means that even if somebody should gain access to the encrypted data on the server and the key used to encrypt it, they would not be able to decrypt it again. It does also mean, however, that if the researchers lose the corresponding key for decryption, they will not be able to decrypt the donated chat logs again. We thus advise keeping the second key pair for decryption offline on a well-secured device. While RSA encryption provides an additional layer of security for participants’ data, we would like to emphasize that it is no substitute for a properly set up and maintained server infrastructure, good research data management, and rigorous testing of the data donation pipeline before participants’ data are processed (see also section “The DashboardTester script”). For a step-by-step guide on how to create key pairs and set up ChatDashboard, please refer to the supplementary materials on our GitHub page.^25^

### Data visualization

After the donated data have been checked, named, encrypted, and stored on the server, participants are forwarded to a page where they can interactively explore their own data (see Appendix Fig. 8). Importantly, this page uses a copy of the originally parsed dataset by default, containing all information present in the raw dataset, including columns that potentially contain PII and parts that have been manually excluded by participants. This enables participants to recognize themselves in the analyses and plots, and to transparently explore all of their own data, even though they might not feel comfortable donating some parts of it. Crucially, though, only participants themselves ever have access to these visualizations. The dataset used for the visualizations is deleted from the server when the session ends (participants close the browser window, time out, or are disconnected), and only the reviewed, anonymized, and encrypted data are persistently stored on the server. Visualizations are created using the plotting functions from the WhatsR package (see Table 2) and made interactive in Shiny by allowing users to set parameters such as timeframe, chat participant names, or minimum number of occurrences in the UI, which passes them on to the plotting functions. By default, ChatDashboard showcases plots for the number of messages, links, smileys, emojis, and reply times, using bar graphs, heatmaps, and cumulative line graphs. However, all plotting functions from the WhatsR package (or custom ones) could be used to expand and adapt the interactive exploration page in the “server” and “ui” parts of the app.R script in ChatDashboard.

### Summary

In sum, ChatDashboard is an R Shiny web app leveraging the functions of the WhatsR R package to enable researchers to build and host their own interactive and transparent WhatsApp chat log donation platforms. It provides participants with a platform to securely upload, transparently review, and select their data for donation, and gives them an opportunity to interactively explore their own chatting behavior. At the same time, the default version of the platform can ensure that researchers only ever have access to reviewed, anonymized, donated, and encrypted data instead of having to interact with the raw, exported chat logs still containing PII. Importantly, though, researchers can fully customize which variables are automatically removed, and can also decide to keep raw chat messages or implement their own anonymization functions by adapting the app.R script.

ChatDashboard can be set up in a way to make donated chat logs linkable to survey responses collected on other platforms through passed URL parameters, and is accessible to social scientists because it is built entirely in R. In the next section, we will discuss the final part of the framework—DashboardTester—an automated script to simulate research participants on ChatDashboard for testing that the data donation platform is working as intended.

## The DashboardTester script

Raw WhatsApp chat logs exported by research participants contain highly personal information by default. Therefore, setting up a data donation platform for WhatsApp chat logs requires thorough testing before data from real participants can be processed. On the one hand, the platform must work well on a usability level, so that interested research participants do not face disconnects, bugs, infinite loading screens, or visualizations that do not display informative graphs. Should this occur, participants might lose motivation to go through with the donation process or lose trust in the researchers or the web app processing their data. We already tested ChatDashboard with students and colleagues to implement their feedback about the user experience. However, there has not yet been any professional testing of the user experience using real study participants or researchers seeking to set up a data collection pipeline for themselves. This kind of testing could be a promising avenue for further improvement and to maximize utilization of the framework and participation rates in studies.

On the other hand, the platform must work well on a technical level so that data are parsed correctly, can only be accessed by research participants themselves in raw format, are subset correctly, variables defined to be excluded are excluded reliably, chat logs are encrypted correctly before storage on the server, and data can be decrypted again by the researcher. This testing process can be extremely tedious because one has to test chat logs exported from different phone operating systems, with different language settings, containing different kinds and amounts of data, different numbers of chat participants, and a variety of different user inputs and behaviors on the data donation platform. While the WhatsR package can simulate a wide variety and large amount of different, simulated chat logs using the create_chatlog() function, manually uploading and acting as a research participant to test ChatDashboard would be a time-consuming and error-prone process. We thus built an automated testing script for simulating participants interacting with ChatDashboard and donating simulated data.

DashboardTester is a collection of R scripts and files to test the correct setup of ChatDashboard by simulating participants signing up, uploading a simulated chat log to the web app, randomly selecting data to be donated, and donating the dataset. While doing so, it logs the actions of simulated participants and the responses of the web app to enable a comparison of the donated datasets with the actions of simulated participants. Through this comparison, researchers can ensure that the data donation is working, variables defined for exclusion are reliably excluded, only selected data are donated, and encryption and decryption of the data are working as intended. In the following sections, we will describe DashboardTester in greater detail. For a detailed guideline on how to run the script yourself, please refer to the supplementary materials on our GitHub page.^26^

### Simulating chat logs

To test chat logs exported from different phone operating systems, different language settings and different time settings, the create_chatlog() function from the WhatsR package (see Table 2) can be used. Specifically, researchers can use the parameters of the function to define what kinds of chat logs, how many, and which combinations of features should be simulated. For a detailed explanation of the parameters, interested researchers can refer to the corresponding documentation by running “?create_chatlog()” in R after installing the WhatsR package. We suggest simulating a sufficient number of chat logs (e.g., 1000), with parameter combinations that correspond as closely as possible to chat logs expected from the intended sample of participants, to maximize the likelihood of catching any errors early during testing. Simulated chat logs should be saved in the “UploadData” folder before running the simulation (see Table 4). We recommend not only testing artificially created chat logs, but also including logs that were recently extracted manually from a chat between two consenting researchers. This ensures that the structure of chat logs has not recently and unannouncedly changed in a way that negatively impacts the function of WhatsR or ChatDashboard (see below).

**Table 4:** Overview of contents and functions of DashboardTester

File/folder	Function	Relative file path
RunningSimulation.R	Code for running simulation of participants on ChatDashboard instance and specifying simulation parameters	./ RunningSimulation.R
SimulateChatDashboardParticipant.R	Script containing the function to simulate a single participant on a ChatDashboard instance using RSelenium. Called by RunningSimulation.R for every simulated participant.	./ SimulateChatDashboardParticipant.R
README.md	Overview of DashboardTester and how to set it up	./README.md
UploadData	Folder for raw chat log data to be uploaded in simulation	./UploadData
AnalyzingSimulation	Folder for necessary files and scripts for analyzing the simulation results	./AnalyzingSimulation/
DecryptionKeypair	Folder for storing RSA keypair necessary for decrypting chat logs encrypted by the ChatDashboard instance during the simulation	./AnalyzingSimulation/DecrpytionKeypair/
SimulatedDonations	Folder for storing encrypted chat logs donated during the simulation	./AnalyzingSimulation/SimulatedDonations /
SimulationLogs	Folder for storing logs of simulated participant behavior returned by RunningSimulation.R	./AnalyzingSimulation/SimulationLogs /
AnalyzingSimulation.R	Checks if any PII or non-selected data is contained in simulated data donations. Also checks if files can be decrypted	./ AnalyzingSimulation/AnalyzingSimulation.R
LICENSE	Terms of the GPL3 license	./LICENSE

### Simulating participants

To simulate participants on the ChatDashboard web app, researchers can run the RunningSimulation.R script. The script allows researchers to define the address of the ChatDashboard instance that is being tested (“url”), a string for naming test participants to distinguish them from real participants (“id”), the password for accessing the platform as specified in the app.R script (“password”), the browser that should be used to simulate participants (“browser”), the version of the browser that should be used (“version”), the port that should be used (“port”), and the path to the “UploadData” folder containing the simulated chat logs used for testing (“filePath”). Importantly, the specified browser and the desired browser version have to be installed on the system of the researcher running the script, the specified port has to be available, and R must have permission to read and write to the specified folders.

When the script is run, it calls the function in SimulateChatDashboardParticipant.R to navigate to the specified instance of ChatDashboard and simulate the behavior of a research participant using the platform. To do so, the script uses functions from the RSelenium package (Harrison, 2022) to open the specified browser, navigate to the ChatDashboard web app, log in with the specified participant id and password, and upload one of the simulated chat log files from the “UploadData” folder. The script then waits for the web app to parse the chat log and display it as a table, and subsequently simulates participant behavior by randomly selecting and deselecting columns, excluding rows, or restoring the initial selection. The result of this process is the selection of a random subset of the uploaded data. After this, the script clicks the “donate data” button and either confirms the current selection for data donation or goes back with a 50% chance of restarting the data selection process. Should the random selection contain any columns defined to be removed, the script automatically clicks on “okay” in the popup message informing participants about the automatic removal of these columns.

After this, the script saves a log of information about the parameters used, all simulated participant actions, the selected columns, excluded rows, and any server-side messages that were created during the interaction with the browser session. The donated, simulated chat log is encrypted and saved in the “UserData” folder of the ChatDashboard directory of the server. This process is executed for the number of simulated participants specified by the “simulate_n_users” parameter in the RunningSimulation.R script and takes between 2 and 3 minutes per simulated participant on our machine. Simulating a substantive number of users can thus take several days.

### Checking results

After the simulation of participants is finished, researchers on the one hand have a log file listing all simulated participants, the data they uploaded, what they clicked on and selected, and all server-side messages generated during this process, and on the other hand, a list of simulated chat logs donated by simulated participants. In a final step, researchers can then download and decrypt the donated simulated chat logs and compare their contents with the generated log files. Specifically, for every donated chat log, researchers should check (1) whether any columns are contained that were defined to be automatically removed, and (2) whether any columns are contained that were excluded by simulated participants. If any data are contained that should have been automatically removed or were not explicitly donated by simulated participants, the platform is not working as intended and should not be deployed to actual research participants until the issue is identified and fixed, and further test runs come back clean. For a detailed step-by-step guideline on how to set up and run DashboardTester, please refer to the supplementary materials on our GitHub page.^27^

### Caveats

We would like to conclude this section with some words of caution. The WhatsR package, ChatDashboard, and DashboardTester were developed by a computational social psychologist and not a team of professional software developers. As such, the software might contain inefficient code or bugs, or might not work correctly on some machines. Should you intend to use the software and encounter any issues, we kindly invite you to report them on our GitHub issues pages so that we can check and potentially address them. Moreover, should researchers adapt or improve on the source code, we kindly invite them to suggest the changes for a merge into the repository via a pull request on GitHub.

Most importantly, however, the parse_chat() function and thus also the anonymization and subsetting features in ChatDashboard are very much dependent on the document structure of exported WhatsApp chat logs (see Kohne et al., 2022). This structure has been changed unannouncedly by Meta in the past and will probably be changed in the future as well. If such a change occurs, and the create_chatlog() function is not adapted accordingly (yet), simulated testing chats will have a different structure from those donated by actual participants. In a worst-case scenario, DashboardTester would then show no issues for simulated participants, while real participants might not be able to donate data, or data might be processed incorrectly. We thus strongly recommend testing ChatDashboard not only with simulated chat logs before deploying it to research participants, but also with a limited number of real chat logs between two consenting co-authors, that were manually exported recently. This can ensure that no recent, unannounced changes to the WhatsApp chat log structure are breaking the functionality of our framework. In the case of any doubts that the data donation platform is working securely and correctly, we always advise to err on the side of caution and to not collect any data from participants until all issues are resolved and tested.

## Summary and outlook

Exported WhatsApp chat logs are a new and unique type of data that allow researchers to quantify human communication and social interactions in unprecedented detail. Furthermore, the widespread use of WhatsApp (Kemp, 2020; Montag et al., 2015) and the feature for users to export their own chat logs make data donations a viable option for collecting data from many different samples of interest. Because data can be extracted retrospectively, and participants are not aware at the time of chatting that they might donate data for research purposes later, exported WhatsApp chat logs can help circumvent common issues with social desirability, memory effects, or experimenter bias that are frequent limitations of other data sources (see section “Instant messenger data”). However, collecting exported and donated chat log data has been a challenge for most social science researchers until now due to several obstacles.

With the ChatDashboard framework presented in this paper, we hope to provide a basis for social scientists to set up their own WhatsApp chat log data collection platforms without the need to build their own infrastructure completely from scratch. The WhatsR R package can be used to import and parse WhatsApp chat logs, extract message features into dedicated columns, and remove PII from the chat logs. Moreover, it can also check for predefined consent messages, and remove content from all chat participants who did not post the consent message into the chat. In addition, it also contains various functions for summarizing and visualizing WhatsApp chat log data. It can be used either as a standalone tool for processing chat log files donated to researchers through other channels, or in combination with ChatDashboard, our R Shiny web app for transparent and interactive data donations.

ChatDashboard allows participants to upload their data into a password-protected environment, interactively explore it, and decide for themselves which parts they are willing to donate. After data donation, participants gain access to interactive visualizations to explore their own communication behavior, which can also serve as an incentive for participation (cf. Schwind & Seufert, 2018; Seufert et al., 2015). Donated data are encrypted and can only be decrypted by the researcher.

To test the correct setup of ChatDashboard, researchers can use the WhatsR package to simulate artificial chat logs, and the DashboardTester script to simulate participants interacting with the website and donating simulated data. All code is freely available under the GPL3 license through our GitHub repositories, so we hope that this paper, as well as the ChatDashboard framework and the corresponding documentation, will make WhatsApp data collections much more accessible to social scientists in the future.

From a substantive perspective, we hope that ChatDashboard will contribute to the exploration, testing, and refinement of current theories about interpersonal processes, relationships, communication, and social influence with highly granular, retrospectively collected behavioral data. This kind of data can provide a window into how people communicate in private about specific topics or events (e.g., Garimella & Tyson, 2018; Jensen & Hussong, 2021), how friendships (e.g., Harari et al., 2020; Underwood et al., 2012) or romantic relationships (e.g., Brinberg et al., 2021; Brinberg & Ram, 2021; Underwood et al., 2012) develop over time, how social groups form (cf. Sprugnoli et al., 2018) and communicate (e.g., Schwind & Seufert, 2018; Seufert et al., 2015, 2016), or even how groups radicalize themselves (Schulze et al., 2022; Urman & Katz, 2022).

From a methodological perspective, we hope that the ChatDashboard framework will be used, but also adapted and improved upon by other researchers. Research infrastructures are too often treated as a zero-sum game, so that scientists have to reinvent the wheel again and again for collecting the same kinds of data. To counteract this, we actively invite other researchers to contribute and improve upon it, so that WhatsApp data can be effectively used in more social science research projects.

## Figures and Tables

**Fig. 3:**
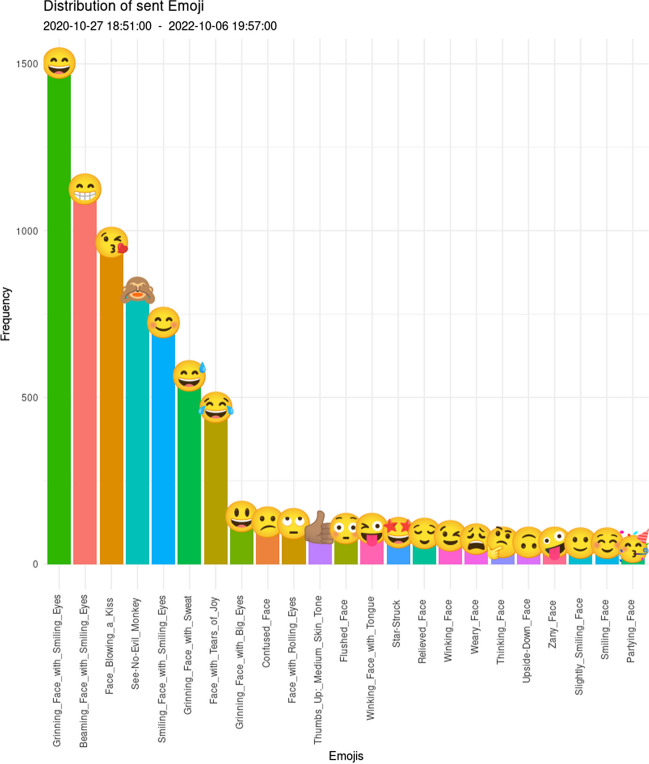
Distribution of emojis sent in a WhatsApp chat, preprocessed with the parse_chat() function and plotted with the plot_emoji() function.

**Fig. 4:**
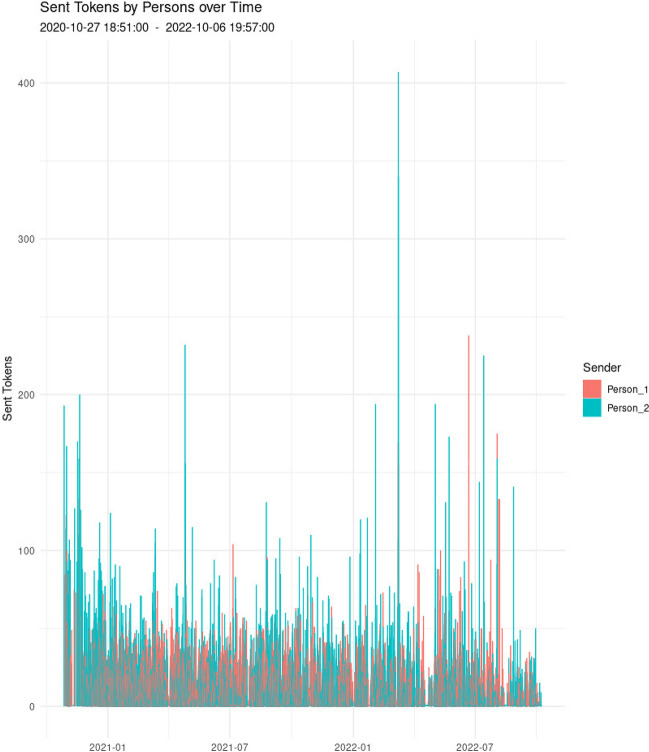
Amount of sent tokens in a WhatsApp chat log per person over time. Data were preprocessed using the parse_chat() function and visualized using the plot_tokens_over_time() function.

**Fig. 5:**
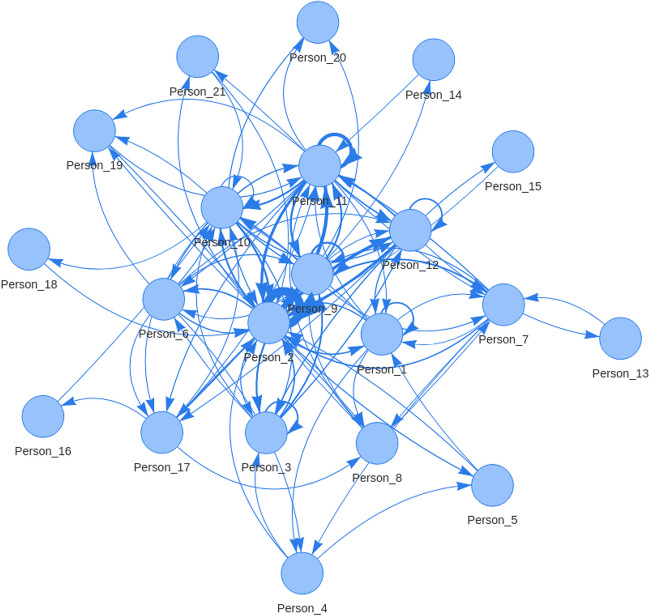
Network of replies from a WhatsApp group chat. Each node represents a group chat participant, each edge represents a reply to a previous message. Data were preprocessed using the parse_chat() function and visualized using the plot_network() function.

**Fig. 6:**
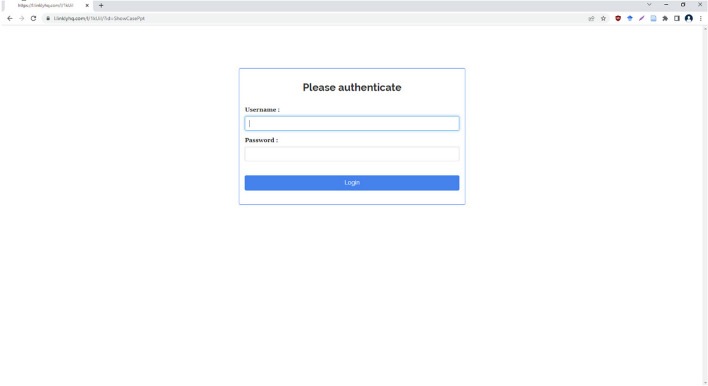
ChatDashboard landing page for authenticating participants. Participants can log in with a valid username and password. These can be either predefined or generated using a passed URL parameter (e.g., "?id=”) to enable linking with anonymous survey responses.

**Fig. 7:**
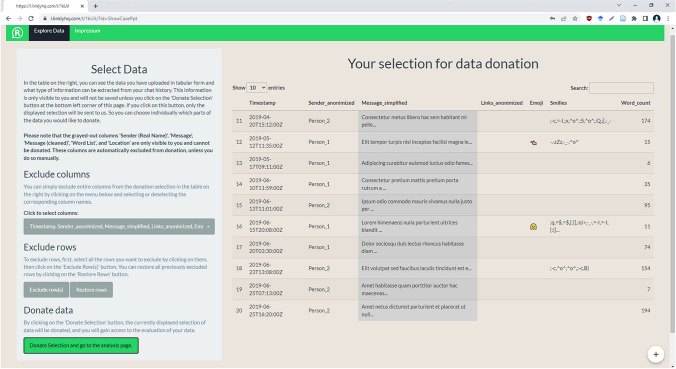
ChatDashboard data selection page. Participants can see a parsed version of their uploaded data and decide interactively which parts they want to donate. Each column and row can be interactively excluded from the donation. Columns containing PII are colored in gray and cannot be donated, even if manually selected.

**Fig. 8:**
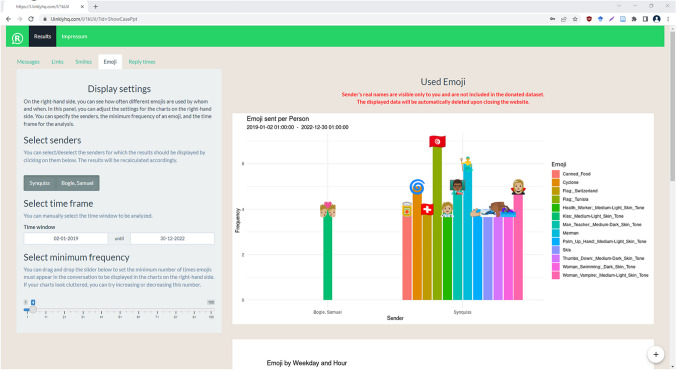
ChatDashboard feedback page. After donating anonymous data, participants get access to interactive visualizations of their own chatting behavior. These display the raw data with all contained PII but are only ever accessible to participants themselves. All raw data and visualizations are deleted as soon as participants close the website. The displayed screenshot is based on simulated data using WhatsR::create_chatlog()

## Data Availability

Data sharing is not applicable to this article, as no datasets were generated or analyzed during the current research project.

## Footnotes

1 See https://faq.whatsapp.com/1180414079177245/?cms_platform=android

2 https://github.com/gesiscss/WhatsR/blob/master/README.md

3 https://github.com/gesiscss/ChatDashboard/blob/master/README.md

4 https://github.com/gesiscss/DashboardTester

5 https://creativecommons.org/licenses/by-nc-sa/4.0/

6 See https://en.wikipedia.org/wiki/Regular_expression

7 See https://en.wikipedia.org/wiki/Left-to-right_mark

8 See https://en.wikipedia.org/wiki/Zero-width_space

9 Indicators for omitted media files are only present in the chat logs if participants selected the “without media” option during export, see also: https://faq.whatsapp.com/1180414079177245/?helpref=uf_share

10 See https://stat.ethz.ch/R-manual/R-devel/library/base/html/as.POSIXlt.html

11 E.g., https://www.google.com/maps/search/?api=1&query=50.94258329999999,6.952524291403516

12 Built from icons listed in https://en.wikipedia.org/wiki/List_of_emoticons

13 See https://faq.whatsapp.com/1180414079177245/?cms_platform=android

14 See https://home.unicode.org/

15 See https://en.wikipedia.org/wiki/Lorem_ipsum

16 See https://shiny.rstudio.com/

17 See https://github.com/gesiscss/ChatDashboard/blob/master/README.md

18 See https://shiny.molekulare-psychologie.de/jkohne/ChatDashboardShowcase/

19 For example, www.UrlToServer.de/?id=TestUser

20 See https://shiny.rstudio.com/articles/tag-glossary.html

21 See https://faq.whatsapp.com/1180414079177245/

22 See https://shiny.rstudio.com/articles/scoping.html

23 See https://en.wikipedia.org/wiki/SHA-2

24 See https://docs.ropensci.org/cyphr/articles/cyphr.html

25 See https://github.com/gesiscss/ChatDashboard/blob/master/README.md

26 See https://github.com/gesiscss/DashboardTester/blob/master/README.md

27 See https://github.com/gesiscss/ChatDashboard/blob/master/README.md
